# HEALTH STATUS AND ENVIRONMENTAL FACTORS ASSOCIATED WITH OLDER ADULTS’ MOBILITY IN 2021 COMPARED TO 2020

**DOI:** 10.1093/geroni/igad104.2373

**Published:** 2023-12-21

**Authors:** Namkee Choi

**Affiliations:** University of Texas, Austin, Texas, United States

## Abstract

The COVID-19 pandemic has had significant negative impact on life-space mobility of people of all ages around the world. Using the 2019-2021 National Health and Aging Trend Study (N=3,063, age 70+) and the socioecological mobility framework, we examined the changes in the frequency of going outside among U.S. older adults between 2020 (during the pandemic) and 2021 (post-COVID vaccine). Respondents self-reported changes in their frequency of going outside: no change (=about the same), increased frequency (=more often), and decreased frequency (=less often). We then fit multinomial logistic regression to examine the associations of the changes in the frequency of going outside with physical, psychosocial, and cognitive health, environmental (COVID concerns and transportation) factors, and social media use, controlling for the 2020 frequency of going outside. We found that in 2021 compared to 2020, 13% and 16% of those age 70+ reported increased and decreased frequencies, respectively. Increased frequency was associated with social media use. Decreased frequency was associated with poor physical health, depression/anxiety, and perceived memory decline. COVID concerns and transportation problems, as well as female gender, age 90+, and being non-Hispanic Black, were also significant correlates of decreased frequency. In conclusion, most U.S. adults age 70+ appear to have resumed their 2019 level of frequency of going outside in 2021 after the COVID vaccines became available; however, 16% reported decreased frequency of going outside in 2021 compared to 2020. Older adults with physical, mental, and cognitive health challenges need help to increase their frequency of going outside.

